# A BAC clone fingerprinting approach to the detection of human genome rearrangements

**DOI:** 10.1186/gb-2007-8-10-r224

**Published:** 2007-10-22

**Authors:** Martin Krzywinski, Ian Bosdet, Carrie Mathewson, Natasja Wye, Jay Brebner, Readman Chiu, Richard Corbett, Matthew Field, Darlene Lee, Trevor Pugh, Stas Volik, Asim Siddiqui, Steven Jones, Jacquie Schein, Collin Collins, Marco Marra

**Affiliations:** 1 BC Cancer Agency Genome Sciences Centre, West 7th Avenue, Vancouver, British Columbia, Canada V5Z 4S6; 2 Cancer Research Institute, University of California at San Francisco, San Francisco, California, USA 94143-0808

## Abstract

We present a method, called fingerprint profiling (FPP), that uses restriction digest fingerprints of bacterial artificial chromosome clones to detect and classify rearrangements in the human genome. The approach uses alignment of experimental fingerprint patterns to *in silico *digests of the sequence assembly and is capable of detecting micro-deletions (1-5 kb) and balanced rearrangements. Our method has compelling potential for use as a whole-genome method for the identification and characterization of human genome rearrangements.

## Abstract 2

Fingerprint Profiling (FPP) is a new method which uses restriction digest fingerprints of bacterial artificial chromosome (BAC) clones for detecting and classifying rearrangements in the human genome.

## Background

The phenomenon of genomic heterogeneity, and the implications of this heterogeneity to human phenotypic diversity and disease, have recently been widely recognized [1-5], energizing efforts to develop catalogues of genomic variation [6-12]. Among efforts to understand the role and effect of genomic variability, landmark studies have described changes in the genetic landscape of both normal and diseased genomes [13-15], the presence of heterogeneity at different length scales [5,16] and variability within normal individuals of various ethnicities [17-19]. Genome rearrangements have been repeatedly linked to a variety of diseases, such as cancer [20] and mental retardation [21], and the evolution of alterations during disease progression continues to be an emphasis of current studies.

Presently, various array-based methods, such as the 32 K bacterial artificial chromosome (BAC) array and Affy 100 K SNP array [21-23], are the most common approaches to detecting and localizing copy number variants, which are one class of genomic variation. The ubiquity of arrays is largely due to the fact that array experiments are relatively inexpensive, and collect information genome-wide. The advent of high-density oligonucleotide arrays, with probes spaced approximately every 5 kb, has increased the resolution of array methods to about 20-30 kb (multiple adjacent probes must confirm an aberration to be statistically significant) [21]. Despite their advantages, commonly available array-based methods have several shortcomings. These include the inability to: detect copy number neutral variants, such as balanced rearrangements; precisely delineate breakpoints and other fine structure details of genomic rearrangements; and directly provide substrates for functional sequence-based characterization once a rearrangement has been detected.

Clone-based approaches have been developed to study genome structure, in part motivated by shortcomings of array-based methods [16,24,25]. In addition to their use in identifying both balanced and unbalanced rearrangements, clones have the potential to be directly used as reagents for downstream sequence characterization and cell-based functional studies [24]. Despite the advantages of clone-based methods, relatively few studies have reported their use for detecting and characterizing genomic rearrangements. End sequences from fosmid clones have been compared to the human reference genome sequence to catalogue human genome structural variation [16]. End sequence profiling (ESP) [25], which uses BAC end sequences, has been used to study genomic rearrangements in MCF7 breast cancer cells [24]. The principal drawbacks of clone-based methods are cost and speed of data acquisition. For example, in the case of end sequencing approaches that sample only the clone's termini, deeply redundant clone sampling would be required to approach coverage of the human genome. This might require millions of clones and end sequences. More tractable might be an approach capable of sampling the entire insert of a clone rather than only the ends, thereby enhancing coverage of the target genome with fewer sampled clones. Clone coverage of the human genome could then be achieved with only a small fraction of the clones required to achieve comparable genome coverage in clone end sequences.

One method for sampling clone inserts is restriction fragment clone fingerprinting, which has been used by us and others to produce redundant clone maps of whole genomes [21,26-30]. Whole-genome clone mapping projects have shown that it is possible to achieve saturation of mammalian genome coverage with 150,000-200,000 fingerprinted BACs, with the number of BACs required inversely proportional to BAC library insert sizes. This relatively tractable number of clones suggests that whole genome surveys using BAC fingerprinting are feasible. What is not known is whether fingerprints are capable of identifying clones bearing genome rearrangements. In this study we address this question using computational simulations and fingerprint analysis of a small number of BAC clones, previously characterized by ESP. We collected restriction enzyme fingerprints from a set of 493 BACs that represented regions of the MCF7 breast cancer cell line genome. Using an alignment algorithm we developed (called fingerprint profiling (FPP)), we fingerprinted clones and aligned these fingerprints to locations on the reference genome sequence and used the alignment profiles to detect candidate genomic rearrangements. Our analysis reveals fingerprint analysis can detect small focal rearrangements and more complex events occurring within the span of a single clone. By varying the number of fingerprints collected for a clone, the sensitivity of FPP can be tuned to balance throughput with satisfactory detection performance. We also show that FPP is relatively insensitive to certain sequence repeats. Our analysis is compatible with the concept of using clone fingerprinting to profile entire genomes in screens for genome rearrangements.

## Results

We explored the utility of FPP for the identification of genome rearrangements. The method involved generating one or more fingerprint patterns by digesting clones with several restriction enzymes, and comparing these patterns to *in silico *digests of the reference human genome sequence. Differences detected in this comparison identified the coordinates of candidate genome rearrangements.

### Restriction enzyme selection

**Figure 1:**
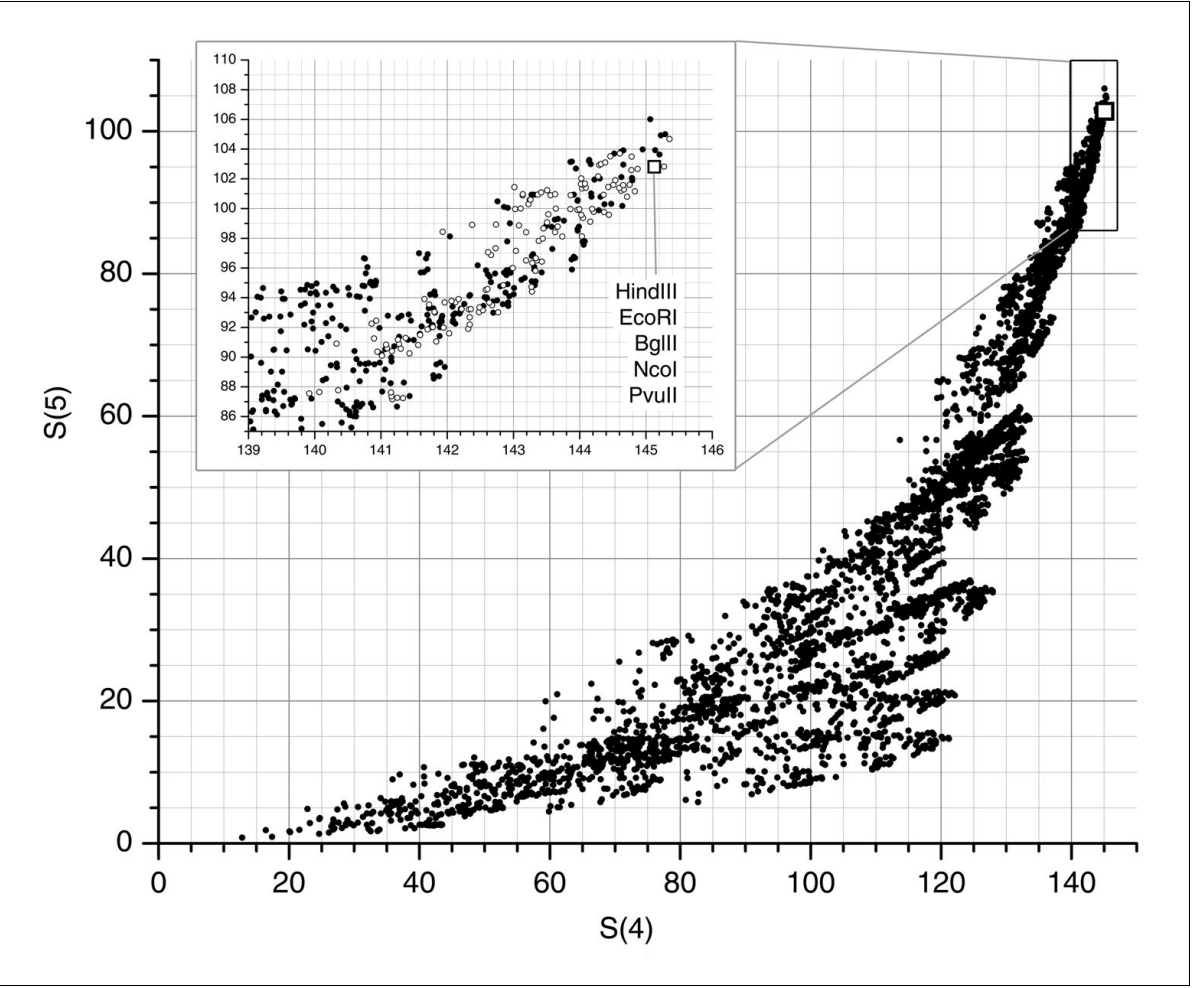
Desirability ranking of 4,060 five-enzyme combinations. We determined desirability of enzyme combinations based on S(n), defined as the fraction of the chromosome 7 that is represented by restriction fragments in the range 1-20 kb (a subset of our sizing range within which sizing accuracy is increased) for ≥n enzymes. Enzyme combinations with high values of S(n) are desirable because a large fraction of fragments in their fingerprint patterns can be accurately sized and because the number of large fragment covers found in regions represented exclusively by large fragments in all digests is minimized. Points represented by hollow glyphs correspond to enzyme combinations which achieved rank in top 10% for *each *of S(*n *= 1..5).

We analyzed the distribution of recognition sequences for 4,060 restriction enzyme combinations (Figure 1) on human chromosome 7 (Materials and methods). From this, we identified five restriction enzyme combinations of potential utility for FPP. All five combinations included *Hin*dIII and *Eco*RI, and one of: *Bcl*I/*Bgl*II/*Pvu*II, *Bal*I/*Bcl*I/*Bgl*II, *Nco*I/*Pvu*II/*Xba*I, *Bcl*/*Nco*I/*Pvu*II, or *Bgl*II/*Nco*I/*Pvu*II. Each of these combinations represented at least 99.98% of the chromosome7 sequence in restriction fragments of sizes that are generally accurately determined using our BAC clone fingerprinting method. Ultimately, we selected the combination *Hin*dIII/*Eco*RI/*Bgl*II/*Nco*I/*Pvu*II for its desirable cut site distribution, ease of use in the laboratory and our favorable experience with the high quality of fingerprints from these enzymes.

### Theoretical sensitivity of fingerprint alignments

To demonstrate that fingerprint patterns are sufficiently complex to uniquely identify genomic intervals, we devised *in silico *simulations to determine specificities of fingerprint fragments and patterns and to align virtual clones with simulated rearrangement breakpoints to the reference genome sequence.

**Figure 2:**
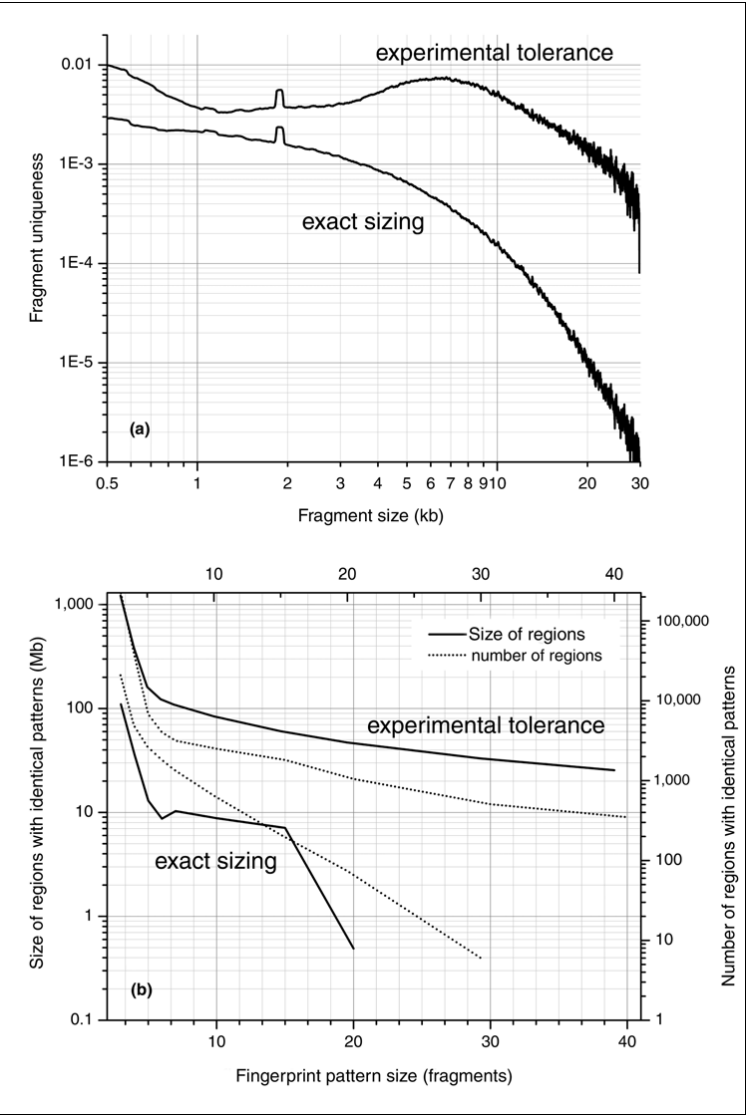
Specificity of individual restriction fragments and patterns based on exact and experimental sizing tolerance. **(a) ***Hin*dIII restriction fragment specificity for the human genome for fragments within the experimental size range of 500 bp to 30 kb. For a given fragment size, the vertical scale represents the fraction of fragments in the genome that are indistinguishable by size in the case of either exact sizing (fragments in common between two fingerprints must be of identical size) or within experimental tolerance (fragments in common between two fingerprints must be within experimental sizing error; Figure 3) on a fingerprinting gel. When sizing is exact, fragment specificity follows approximately the exponential distribution of fragment sizes and spans a range of 3.5 orders of magnitude. When experimental tolerance is included, the number of distinguishable fragment size bins is reduced and the range of fragment specificity drops to two orders of magnitude. **(b) **The specificity of a fingerprint pattern of a given size in the human genome. Fingerprint pattern size is measured in terms of number of fragments. Regions with identical patterns are those in which there is a 1:1 mapping within tolerance between all sizeable fragments. The specificity of experimental fingerprint patterns is cumulatively affected by specificity of individual fragments. The specificity of fragments is sufficiently low (that is, due to high experimental precision) so that 96.5% of the genome is uniquely represented by fragment patterns of 8 fragments or more.

**Figure 3:**
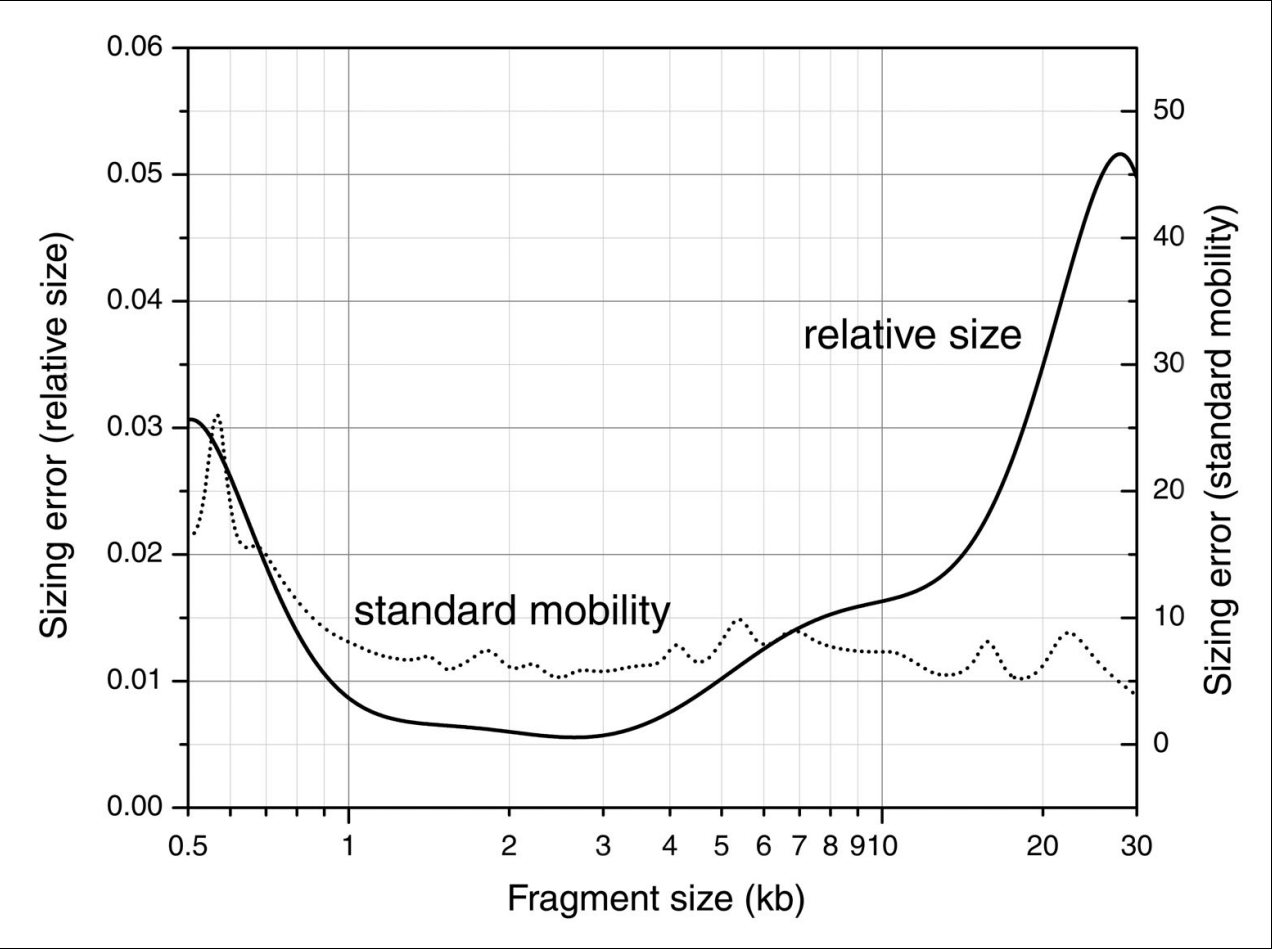
Experimental error of fragment sizing within the 0.5-30 kb sizing range of our single digest protocol. The error is expressed in relative size (left axis) and standard mobility (right axis). Standard mobility is a distance unit that takes into account inter-gel variation and is approximately linear with the distance traveled by the fragment on the gel.

We computed the fragment specificity for a given fragment as the fraction of fragments in the genome that are experimentally indistinguishable in size (Materials and methods). Figure 2 shows the specificity for an individual *Hin*dIII fragment of a given size in the human genome (hg17), and depicts the practical specificity where experimental sizing error is used to determine whether fragment sizes can be distinguished. Our sizing error depends on fragment size (Figure 3), effectively dividing the sizing range into approximately 380 unique bins. Also depicted is the case of exact sizing, where fragments are considered indistinguishable only if their sizes are identical. Although exact sizing is not possible in the laboratory, we include the case of exact sizing here because it represents the theoretical best possible performance of FPP with the enzymes we selected, and because it helps to contrast FPP's practical performance.

This analysis revealed that *Hin*dIII fingerprints with approximately 15 fragments exhibit a high degree of specificity, as only approximately 1.5% of the genome cannot be uniquely distinguished using patterns composed of this number of fragments. This high specificity results from accurate experimental fragment sizing, and from the fact that the length of genomic repeats is generally much shorter than restriction fragments. Therefore, a specific combination of adjacent fragment sizes represents a relatively unique event in the human genome.

**Figure 4:**
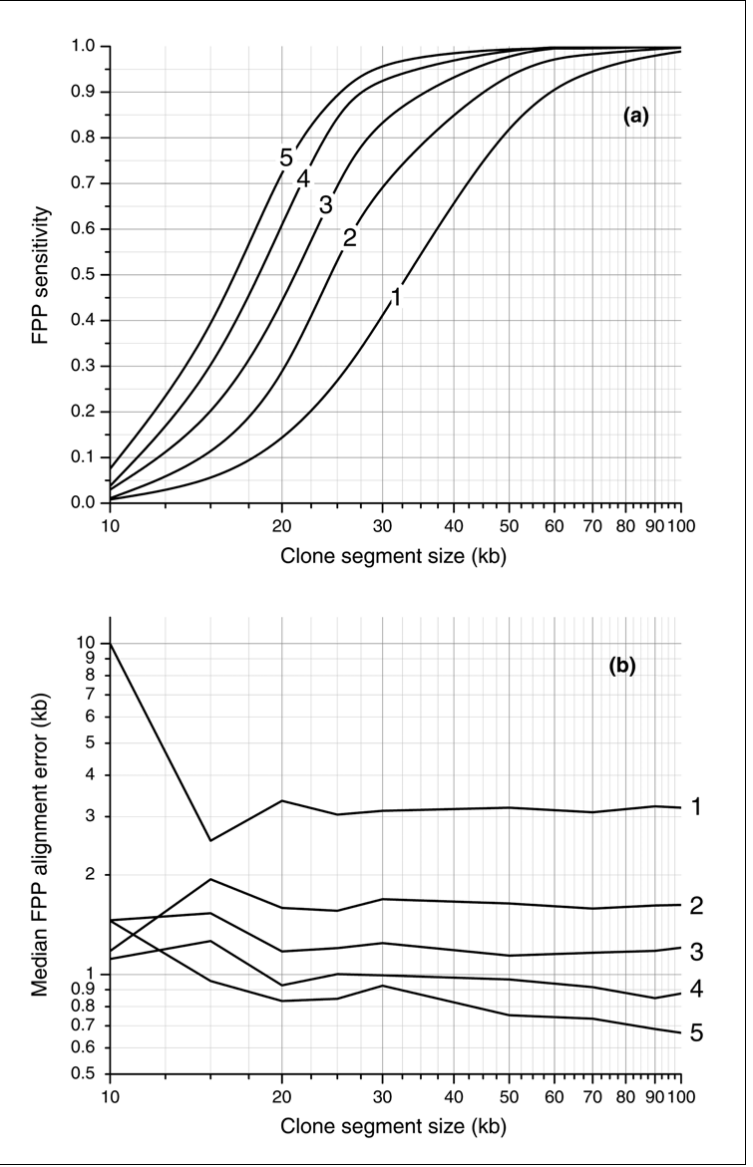
Simulation results of sensitivity and spatial error of rearrangement detection by FPP using experimental sizing tolerance. **(a) **Sensitivity is measured as the fraction of clone regions of a given size with successful FPP alignments and is plotted for five digests (labeled 1-5). **(b) **Spatial error is measured by the median distance between FPP and theoretical alignment positions. The largest improvement in both sensitivity and spatial error is realized by migrating FPP from one digest to two. With two fingerprint patterns used to align the clone, 50% of >25 kb clone regions are aligned (90% of >45 kb regions) with a spatial error of 1.7 kb.

To evaluate the accuracy and sensitivity of actual fingerprint alignments, we performed an *in silico *study (Materials and methods), in which we computationally generated virtual clones containing simulated genomic rearrangement breakpoints and used these fingerprints as inputs into the alignment algorithm. Figure 4 illustrates the sensitivity and positional accuracy of the mapping of these synthetic clones as a function of the number of digests and segment size. When a single *Hin*dIII fingerprint digest is used, we successfully aligned 50% of 35 kb segments. This cutoff size can be decreased to 25 kb if two digests are used (*Hin*dIII/*Eco*RI) and to 16 kb if five digests are used (*Hin*dIII/*Eco*RI/*Bgl*II/*Nco*I/*Pst*II). The number of digests used has a large impact on the smallest alignable segment size due to the fact that the positions of cut sites of distinct enzymes are generally uncorrelated and that the individual digest patterns can be aligned independently and used together to increase sensitivity. Figure 4 suggests the number of digests that would be required to detect 90% of rearrangements of a certain size. For example, if we wish to identify a breakpoint in 90% of simulated cloned rearrangements, then the shortest rearrangements that can be detected for 1, 2, 3, 4 and 5 digests are 60, 45, 34, 28, and 25 kb, respectively. Stated differently, one can be 90% certain that when using 5 enzymes, a segment of length 25 kb within a BAC would be sufficient to identify the BAC as bearing a genome rearrangement.

Figure 4 shows the median distance between the left and right edges of the alignment and known segment spans for segments of varying sizes. While the values for 10 kb segments are difficult to interpret because of relatively few successful alignments, the error is otherwise constant for segment sizes and depends primarily on the number of digests. The error is 3.0 kb for an alignment based on a single digest and drops to 1.7 kb when two digests are used. When the number of digests is increased to 5, the error drops as low as 700 base-pairs (bp).

### MCF7 clone fingerprint-based alignments

With knowledge gained from our simulations, we sought to apply FPP to a test set of 493 BAC clones derived from the MCF7 breast cancer cell line. Each clone was fingerprinted and aligned to the genome with FPP, and the results of the alignments were compared to alignments performed using BAC end sequences (Materials and methods, Additional data file 2). Alignments were evaluated based on their size and number, with multiple alignments indicating identification of a candidate rearrangement. We were able to obtain FPP alignments for 487/493 of the clones. On average, we were able to map 88% of a clone's fingerprint fragments to the genome, and 90% of clones had more than 72% of their fingerprint fragments mapped. Table 1 summarizes FPP and ESP rearrangement detection and Table 2 shows a detailed comparison of rearrangement detection for clones that had an FPP alignment that indicated a breakpoint. The positional accuracy of FPP alignments is shown in Table 3.

**Table 1:** Comparison of number of rearrangements detected by ESP and FPP in a 487 MCF7 BACs

		ESP					
		
		N			Y		
			
		No. of clones	Agree	Disagree	No. of clones	No. agree	No. disagree
FPP	N	250	243	2^b^/5^c^	72	3	63^d^/6^e^
	Y^a^	11	8	2^f^/1^g^	154	126	26^h^/2^i^

The clones are partitioned based on whether a rearrangement was detected by ESP and/or FPP. For each combination of detection (for example, FPP = Y, ESP = N, where Y/N indicates the presence/absence of rearrangement, respectively, as measured by the corresponding method), the table shows the number of clones in this category, which is further broken down into the number of clones in which ESP and FPP mappings agreed and the number of clones for which ESP and FPP mappings did not agree (for example, both can show no rearrangement but disagree about clone position). Clones in the 'Agree' column have an FPP alignment within 50 kb of both end sequence alignments. Clones in the 'Disagree' column are reported as two groups: clones with an FPP alignment agreeing with one end sequence alignment and clones for which no agreement with either end sequence alignment was detected. Both groups with the disagree category are annotated with a reason for the disagreement. ^a^Clones in this row are further classified based on the number of FPP alignments in Table 2. ^b^Del (2); ^c^mispick (5); ^d^bne (33), hr (14), lowcomplex (1), nip (10), rep (5); ^e^lowcomplex (1), mispick (3), rep (2); ^f^rep (2); ^g^mispick (1); ^h^bne (14), hr (8), nip (3), rep (1); ^i^bne (1), mispick (1). Bne, breakpoint near end of clone; del, clone appears deleted; hr, highly rearranged; lowcomplex, fingerprint has very few fragments; mispick, FPP/ESP data mismatch; nip, FPP alignment detected but not added to partition; rep, alignments in repeat regions.

**Table 2:** Profile of candidate rearrangements detected by FPP

		ESP					
		
		N			Y		
			
		No. of clones	Agree	Disagree	No. of clones	No. agree	No. disagree
FPP alignments	2	11	8	1/2	123	101	22/0
	3	0	-	-	29	22	5/2
	4	0	-	-	2	2	0/0

Clones are grouped in rows by the number of distinct FPP alignments. For each group, the clones are partitioned based on whether ESP detected a rearrangement. Clones in the 'Agree' column have an FPP alignment within 50 kb of both end sequence alignments. Clones in the 'Disagree' column are partitioned in the same manner as in Table 1.

**Table 3:** Positional accuracy of FPP alignments

|FPP-BES|	Clone ends*	Clones^†^
<1 kb	50%	28%
<2 kb	70%	50%
<5 kb	88%	79%
<10 kb	96%	93%
<25 kb	99%	98%
<50 kb	100%	100%

Accuracy was measured by comparing the distance between the positions of end sequence alignments and nearest edge of an FPP alignment. For this comparison the subset of clones for which ESP and FPP agreed in both rearrangement detection and mapping position (243 + 126 = 369 clones; Table 1) was used. *Cumulative distribution of nearest distances between FPP and individual BES alignments, min_i_|FPP_i_-BES|. ^†^Cumulative distribution of max_j_(min_i_|FPP_i_-BES_j_|) - the larger of two distances between a clone's FPP and BES alignments

**Figure 5:**
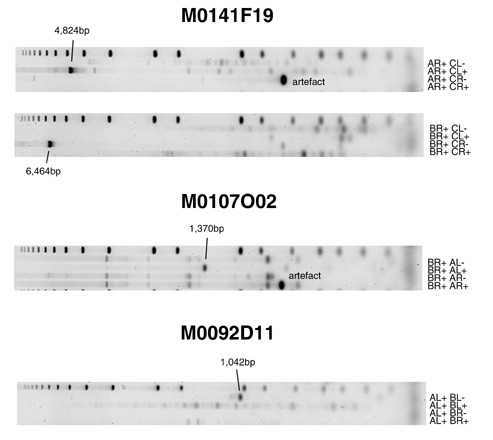
PCR reactions validating the presence of breakpoints in clones listed in Table 5. Each reaction is labeled by the primer combination (e.g. AR+ CL+) used to test order and orientation of the clone's fused regions (Materials and methods; primer combination nomenculature is described in detail in Additional data file 1). The presence of a product demonstrates the adjacency of the regions within the clone's insert.

Because ESP uses BAC end sequences that produce data for only the ends of clones, ESP has limited capacity to localize the locations of rearrangement breakpoints within clones. To investigate the precision of FPP in defining the position of breakpoints within BACs, we used clone alignments spanning regions of chromosomes 1, 3, 17 and 20 that contained known breakpoints. We selected these regions because of the enriched coverage provided by our test clone set. The breakpoint position was determined to be the average FPP alignment position with the error given by the standard deviation of the alignments. Additional data file 2 shows the layout of these breakpoints in the MCF7 genome and all FPP and ESP alignments for clones in these regions. Additional data file 3 expands several of the regions from Additional data file 2, and illustrates the relative position of FPP and ESP alignments. Additional data file 6 further increases the detail shown in Additional data file 2, depicting restriction maps and fragment matching status within each clone alignment for all five enzymes. We found 51 breakpoints in 118 unique clones (Table 4). We tested the presence of breakpoints in three clones using PCR (Table 5), and demonstrated the presence of PCR products (Figure 5) to verify fusions within the clone's insert of regions non-adjacent in the reference genome sequence.

**Table 4:** Location of breakpoints in the MCF7 genome in regions sampled by clones on chromosomes 1, 3, 17 and 20

ID	Chromosome	Position	Uncertainty	Clones
1L	1	106446622		M0035E03
2L	1	107325668	0	M0090F09 M0095D18
3R	1	107642673	1,640	M0012O05 M0064A13 M0089C03 M0090K07 M0126M04 M0152M23
4L	1	112083301	957	M0035A16 M0039B19 M0041G20 M0043K05 M0062P11 M0078P07 M0080G18 M0086B04 M0086C02 M0090F09 M0091L21 M0168M09
5R	1	112119925	0	M0090F09 M0095D18
6R	3	62612471	856	M0012A19 M0041A24
7L	3	63679826	757	M0005P04 M0007J14 M0030P20 M0043O24 M0093C20 M0134N23 M0143D18 M0150I03 M0156K22
8R	3	63716623	1,755	M0005P04 M0007J14 M0030P20 M0043O24 M0093C20 M0107G11 M0134N23 M0137G17 M0143D18 M0150I03 M0151M05 M0156K22
9R	3	63908884		M0035E03
10L	3	63954937	8,740	M0007J14 M0030P20 M0037J18 M0043O24 M0066M03 M0067H12 M0073I23 M0093C20 M0107G11 M0124I19 M0134N23 M0137G17 M0143D18 M0150I03 M0151M05 M0156K22
11R	3	63995878	0	M0066M03 M0067H12 M0124I19 M0137G17
12L	3	63997257	1,178	M0003F05 M0031O08 M0039A05 M0088O13 M0145B06
13R	3	64074753	3,228	M0014E11 M0031O08 M0088O13 M0144L06 M0145B06
14L	3	64660949	0	M0012A19 M0041A24
15R	3	64927120	304	M0006B19 M0014P03
16L	17	54050256	11,312	M0037J18 M0066C22
17R	17	54158022	0	M0037J18 M0073I23
18L	17	54397666	9,801	M0035A16 M0039B19 M0041G20 M0043K05 M0062P11 M0078P07 M0080G18 M0086B04 M0086C02 M0090F09 M0090P15 M0091L21 M0095D18 M0168M09
19R	17	54549098	6,065	M0009I10 M0013G05 M0105A20 M0107H09
20L	17	55260098	5,548	M0001M18 M0009I10 M0013G05 M0107H09
21R	17	55468383	15,761	M0001M18 M0090P15 M0092G06
22L	17	56176919	163	M0089C03 M0090K07 M0126M04 M0152M23
23R	17	56206584	1,204	M0064A13 M0089C03 M0090K07 M0126M04 M0152M23
24R	17	56233933	3,684	M0005P04 M0007J14 M0030P20 M0043O24 M0093C20 M0134N23 M0143D18 M0150I03
25L	17	56644007	1,148	M0005I19 M0045E13 M0054A01 M0054C03 M0058D14 M0058K11 M0059J17 M0062L13 M0077L13 M0089F05 M0089I18 M0094M14 M0107O02 M0124A06 M0132D17 M0138H21 M0145N09 M0147K12 M0148L05 M0159O13 M0160H16 M0165D22
26L	17	56961440		M0021C24
27R	17	57339860	1,364	M0024G06 M0123G10 M0155O05 M0156I16
28L	17	59745950	6,571	M0006B19 M0014P03
29R	17	59781552	688	M0006B19 M0014P03
30L	20	38948829		M0011K13
31L	20	40249289	2,622	M0003F05 M0031O08 M0039A05 M0043G01 M0145B06
32R	20	40271873	1,207	M0003F05 M0031O08 M0039A05 M0043G01 M0088O13 M0145B06
33R	20	40664609		M0011K13
34L	20	45230184	278	M0001A11 M0010D13 M0026L11 M0028H13 M0031E14 M0038G05 M0038P15 M0041B14 M0055I11 M0080H12 M0108H05 M0129A15 M0135D20 M0151F12 M0162M24 M0167J20
35L	20	45736731		M0021C24
36L	20	45847023	1,846	M0014E11 M0088O13 M0144L06
37L	20	46174956		M0159C23
38L	20	48694494	933	M0001A11 M0055I11 M0151F12
39L	20	48729868	6,077	M0010D13 M0026L11 M0028H13 M0031E14 M0038G05 M0038P15 M0041B14 M0080H12 M0108H05 M0129A15 M0135D20 M0162M24 M0165D22 M0167J20
40R	20	48863824	720	M0001A11 M0005I19 M0045E13 M0054A01 M0054C03 M0058D14 M0058K11 M0059J17 M0062L13 M0069H04 M0077L13 M0089F05 M0089I18 M0094M14 M0107O02 M0124A06 M0132D17 M0138H21 M0145N09 M0147K12 M0148L05 M0159O13 M0160H16 M0165D22
41L	20	51618225	4,895	M0003F05 M0005H09 M0008J22 M0029C09 M0031O08 M0036L24 M0043G01 M0071O17 M0075M20 M0077H17 M0090K04 M0100O14 M0116C01 M0132B21 M0145O12 M0159P14
42R	20	52046458	2,367	M0066M03 M0067H12 M0124I19 M0137G17
43R	20	52066649	126	M0012O05 M0089C03 M0152M23
44R	20	52248474		M0066C22
45R	20	52985221		M0014P03
46R	20	53545530	0	M0036B13 M0141F19
47L	20	55122587	853	M0024G06 M0123G10 M0155O05 M0156I16
48L	20	55254895	3,310	M0003F05 M0031O08 M0036L24 M0039A05 M0043G01 M0071O17 M0132B21 M0145B06 M0159C23
49R	20	55287488	1,269	M0003F05 M0005H09 M0008J22 M0029C09 M0031O08 M0036L24 M0039A05 M0043G01 M0071O17 M0075M20 M0077H17 M0090K04 M0100O14 M0116C01 M0132B21 M0145B06 M0145O12 M0159C23 M0159P14
50L	20	59150999	936	M0036B13 M0141F19
51R	20	59176749	0	M0036B13 M0141F19

Breakpoint position is the average position of blunt alignment ends with the standard deviation of these quantities taken as the uncertainty. Breakpoint ID is composed of a unique numerical index and L/R suffix that indicates which edge of the FPP alignment (left/right) is considered to be the breakpoint.

**Table 5:** PCR primers used to validate the presence of breakpoints detected by fingerprints

	Left primer				Right primer			
		
Primer transform	Sequence		Position		Sequence		Position	
		Chr	Start (bp)	End (bp)		Chr	Start (bp)	End (bp)

**M0092D11**								
ar+ br+	TGCTAAATTTCCCAAGTGCC	20	45,794,352	45,794,371	CCGTCCTCTTAGCGAACTTG	20	46,968,304	46,968,323
ar+ br-	TGCTAAATTTCCCAAGTGCC	20	45,794,352	45,794,371	AATTTCAAAATGCGTCTGGG	20	46,968,631	46,968,650
ar+ bl+	TGCTAAATTTCCCAAGTGCC	20	45,794,352	45,794,371	TGACACGCAGGGTAGATCAG	20	46,923,060	46,923,079
ar+ bl-	TGCTAAATTTCCCAAGTGCC	20	45,794,352	45,794,371	TCCAACAGGAAGGAGTACCG	20	46,922,743	46,922,762
al+ br+	CTCTCTTTTGTGGGACGAGC	20	45,718,752	45,718,771	CCGTCCTCTTAGCGAACTTG	20	46,968,304	46,968,323
al+ br-	CTCTCTTTTGTGGGACGAGC	20	45,718,752	45,718,771	AATTTCAAAATGCGTCTGGG	20	46,968,631	46,968,650
al+ bl+	CTCTCTTTTGTGGGACGAGC	20	45,718,752	45,718,771	TGACACGCAGGGTAGATCAG	20	46,923,060	46,923,079
**al+ bl**-	CTCTCTTTTGTGGGACGAGC	20	45,718,752	45,718,771	TCCAACAGGAAGGAGTACCG	20	46,922,743	46,922,762
**M0107O02**								
**br+ ar+**	AATAGAAGCCAGGCATGGTG	20	48,861,156	48,861,175	GTTAGGAGGAGGGTGGAACC	17	56,663,181	56,663,200
br+ ar-	AATAGAAGCCAGGCATGGTG	20	48,861,156	48,861,175	TAGCCGTTCTGACTGGTGTG	17	56,663,261	56,663,280
br+ al+	AATAGAAGCCAGGCATGGTG	20	48,861,156	48,861,175	TAGCTGGGATTACAGGTGCC	17	56,646,379	56,646,398
br+ al-	AATAGAAGCCAGGCATGGTG	20	48,861,156	48,861,175	ACAACCTGTCCGACCAGAAC	17	56,646,305	56,646,324
**M0141F19**								
ar+ cr+	GGACAGAGGCTTTTGTAGCG	17	56,687,628	56,687,647	ACCACGTAGACAAAGACGGG	20	59,173,964	59,173,983
ar+ cr-	GGACAGAGGCTTTTGTAGCG	17	56,687,628	56,687,647	TTCTGGATTCTCCTTGGTGC	20	59,173,950	59,173,969
**ar+ cl+**	GGACAGAGGCTTTTGTAGCG	17	56,687,628	56,687,647	ATTTGGTTCCTGGTGAGTGC	20	59,153,746	59,153,765
ar+ cl-	GGACAGAGGCTTTTGTAGCG	17	56,687,628	56,687,647	AGAAGAACCCGACGACATTG	20	59,153,849	59,153,868
br+ cr+	TATCCTTCAGGAATCGCCAC	20	53,542,992	53,543,011	ACCACGTAGACAAAGACGGG	20	59,173,964	59,173,983
**br+ cr-**	TATCCTTCAGGAATCGCCAC	20	53,542,992	53,543,011	TTCTGGATTCTCCTTGGTGC	20	59,173,950	59,173,969
br+ cl+	TATCCTTCAGGAATCGCCAC	20	53,542,992	53,543,011	ATTTGGTTCCTGGTGAGTGC	20	59,153,746	59,153,765
br+ cl-	TATCCTTCAGGAATCGCCAC	20	53,542,992	53,543,011	AGAAGAACCCGACGACATTG	20	59,153,849	59,153,868

Primer sequence is the appropriately transformed (reversed, complemented, reverse-complemented) primer sequence to test a specific order/orientation of clone regions within the insert. Products were detected for reactions where the primer transform field is in bold. Primer combinations (e.g. ar+ br+) correspond to order and orientation of putative rearrangement and are described in detail in Additional data file 1.

**Figure 6:**
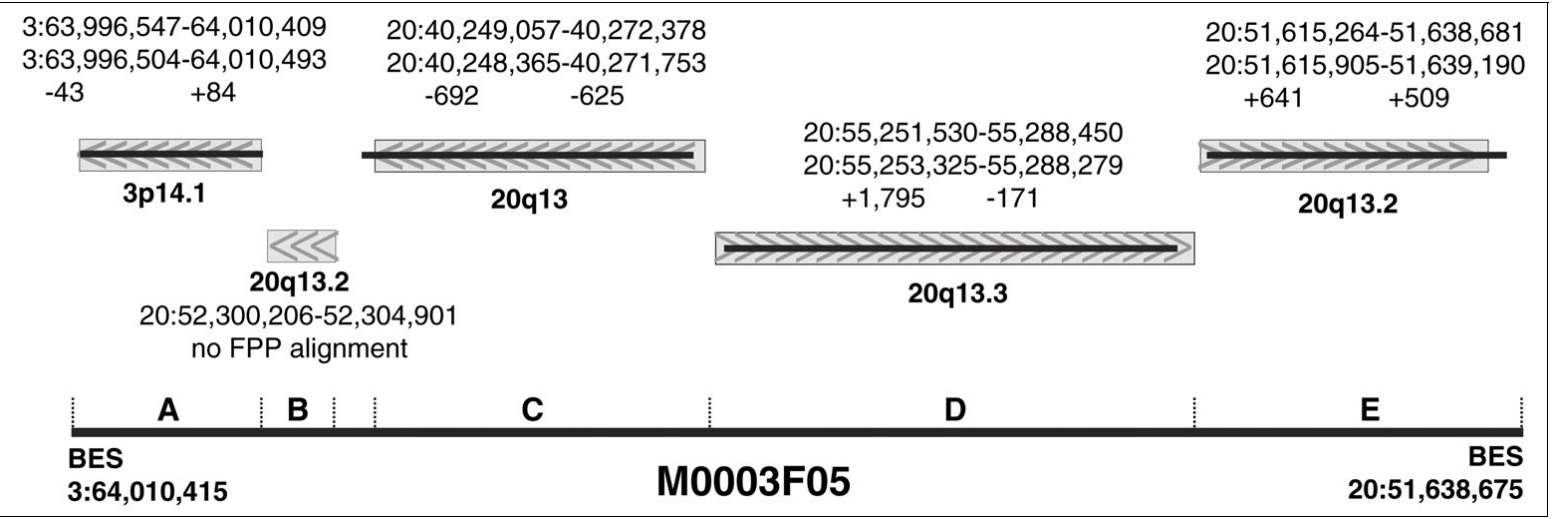
Detailed reconciliation of sequence and fingerprint alignments for clone 3F05, which contains at least four internal breakpoints. FPP is capable of dissecting complex rearrangements in a clone, as illustrated in this figure showing the internal structure of M0003F05. This BAC was sequenced [26] and found to be composed of content from at least five distinct regions (A-E). FPP detected 4/5 of these regions. BLAT (grey rectangles with alignment orientation arrows) and FPP (thin black lines) alignments of M0003F05 are shown; values underneath coordinate pairs are differences in edge positions between BLAT and FPP alignments.

To demonstrate that FPP can resolve complex rearrangements, we closely examined the FPP results for clone 3F5. In the original MCF7 ESP analysis, Volik *et al*. [25] concluded that the shotgun sequence assembly of this clone is highly rearranged and composed of five distant regions of chromosomes 3 and 20 (3p14.1, 20q13.2, 20q13, 20q13.3 and 20q13.2). Our FPP analysis generally recapitulated the shotgun sequencing results - out of the five distinct insert segments found by sequencing, we detected four (Figure 6; detailed fingerprint alignments are shown in Additional data file 4; individual restriction fragment accounting is shown in Additional data file 5). The fifth segment, sized at 4,695 bp based on alignment of the clone's sequence to the reference genome, lacked the fragment complexity to confidently identify it by FPP. This small segment includes only two entire restriction fragments (marked with asterisks in the following list of intersecting fragments) in the restriction map of our enzyme combination (*Hin*dIII, 1 fragment (7.4 kb); *Eco*RI, 3 fragments (7.2 kb, 0.9 kb*, 8.5 kb); *Bgl*II, 2 fragments (4.1 kb, 8.6 kb); *Nco*II, 3 fragments (2.0 kb, 1.9 kb*, 6.2 kb); *Pvu*II, 2 fragments (5.8 kb, 13.1 kb)).

### Micro-rearrangements

**Figure 7:**
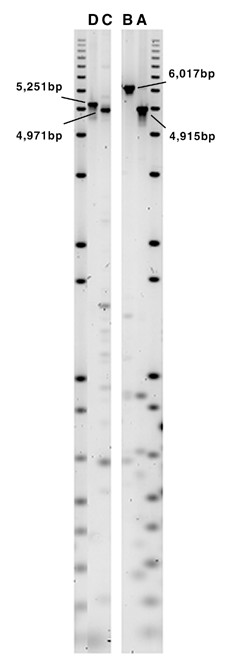
PCR reactions validating small-scale aberrations listed in Table 6. Reactions are labeled A-D, corresponding to the aberrations with the same label in Table 6. In each case the observed product sizes, shown here, are different from the expected sizes based on the inter-primer distance on the reference sequence.

Fingerprints provide a representation of the entire length of a clone's insert and, thus, are capable of mapping genome rearrangements internal to the clone insert that do not involve the ends of the clone. We identified 17 such small-scale candidate aberrations, and validated 4 of these using PCR (Table 6, Figure 7). PCR analysis of clone 12G17 yielded an amplicon approximately 400 bp smaller than expected, which supports the observation that experimental fragments were approximately 300 bp smaller than expected in this area. The fingerprint results are consistent with a hypothesis of a loss of a 313 bp SINE element evident in the genome sequence for this region. PCR analysis of clone 15O22 indicated an insertion of approximately 560 bp relative to the reference genome sequence. The experimental fragments nearest to the unmatched *in silico *fragments in this clone's fingerprints are all about 300 bp larger than expected. The results are consistent with a hypothesis of increased copy number of Alu (300 bp) or SINE (100 bp) elements evident in the genome sequence of this region.

**Table 6:** Location of 17 putative small-scale aberrations identified in MCF7 clones

Aberration position and size						PCR validation		
			
Chr.	Start (bp)	End (bp)	Size (bp)	Affected/all clones	Sampled clone*	Reaction^†^	Primers	Products (bp)
1	54,737,944	54,742,444	4,500	1/1	M0025G14			
2	15,468,892	15,471,992	3,100	1/1	M0015O22	D	GGGGCCCTTTAGTGCCTTAG AATTGCCAAGTCAGAGGCAG	**4,686** 5,251 (+565)
2	110,086,572	110,101,972	15,400	1/1	M0006P20			
3	63,591,911	63,594,011	2,100	2/5	M0118E13			
3	159,597,920	159,602,020	4,100	1/1	M0012G17	B	TACTTACGGCAGAGGTTGGG TCTGATTTTGGAGCTTTTGG	**6,411** 6,017 (-394)
4	13,455,944	13,464,544	8,600	1/1	M0004J18			
5	177,652,902	177,661,002	8,100	1/1	M0019C11			
10	45,658,295	45,662,695	4,400	1/1	M0021J21			
18	13,660,940	13,670,040	9,100	1/1	M0040N18			
19	46,075,421	46,081,021	5,600	1/1	M0005H04			
20	8,877,965	8,903,965	26,000	1/1	M0013M22	A	CTTGGGTTGGGAACTGAAAG CCTCTTCTGGGACTGCTGAC	**28,006** 4,925 (-23,081)
20	39,042,929	39,047,029	4,100	1/1	M0011K13			
20	48,823,455	48,827,555	4,100	3/21	M0107O02			
20	51,886,035	51,891,935	5,900	1/1	M0089C13			
20	52,157,503	52,161,003	3,500	1/1	M0004L22			
20	59,158,037	59,163,037	5,000	1/2	M0141F19			
X	97,281,472	97,287,472	6,000	1/1	M0018J12	C	CCCACCAATGGATTACAACC CTTGAACCTGGGAAGCAGAG	**7,828** 4,971 (-2,857)

Four aberrations were tested with PCR using the primers shown here. The expected primer products based on inter-primer distance on the reference genome are shown in bold, with the observed product sizes shown below. *See Additional data file 2. ^†^See Figure 7.

## Discussion

Using computational simulations and restriction fingerprinting of a small number of BAC clones, we assessed the utility of clone fingerprints in detecting genomic rearrangements. We fingerprinted 493 BAC clones derived from the MCF7 breast cancer cell line genome that were previously analyzed by ESP [25]. Using the clone fingerprints, we aligned the clones to the reference genome sequence assembly (UCSC, hg17) and have mapped the candidate positions of 51 rearrangement breakpoints and 17 micro-rearrangements within clones in the set. Further, we identified other rearrangement events within the clone set that were cryptic to ESP.

The use of fingerprints to detect rearrangements provides several advantages, based on the fact that fingerprints sample essentially all of a clone's insert. First, at equivalent sampling depths, the position of a rearrangement breakpoint within a clone can be more precisely determined using FPP than with ESP. Second, fingerprint patterns can be used to locate differences internal to the insert between the clone and the reference genome. This advantage, which is not shared by ESP (Additional data files 2 and 3), can be leveraged to detect small rearrangements such as single nucleotide polymorphisms, micro-deletions, micro-insertions or other local rearrangements. There is currently no experimental method that can be applied on a whole-genome level that is sensitive to the identification of both balanced and unbalanced rearrangements on the order of 1-5 kb in size within the genome. While extremely high-density oligonucleotide arrays can, in principle, detect aberrations with a spatial frequency equal to probe spacing, confirmation of multiple adjacent probes are required to assign statistical significance to the result. Finally, a major strength of fingerprint alignments is their relative insensitivity to sequence repeats. Although approximately 50% of the human genome sequence assembly (hg17) lies in repeat regions, only 7% is found in contiguous repeat units longer than 3.9 kb, which is the average sizeable *Hin*dIII restriction fragment.

**Figure 8:**
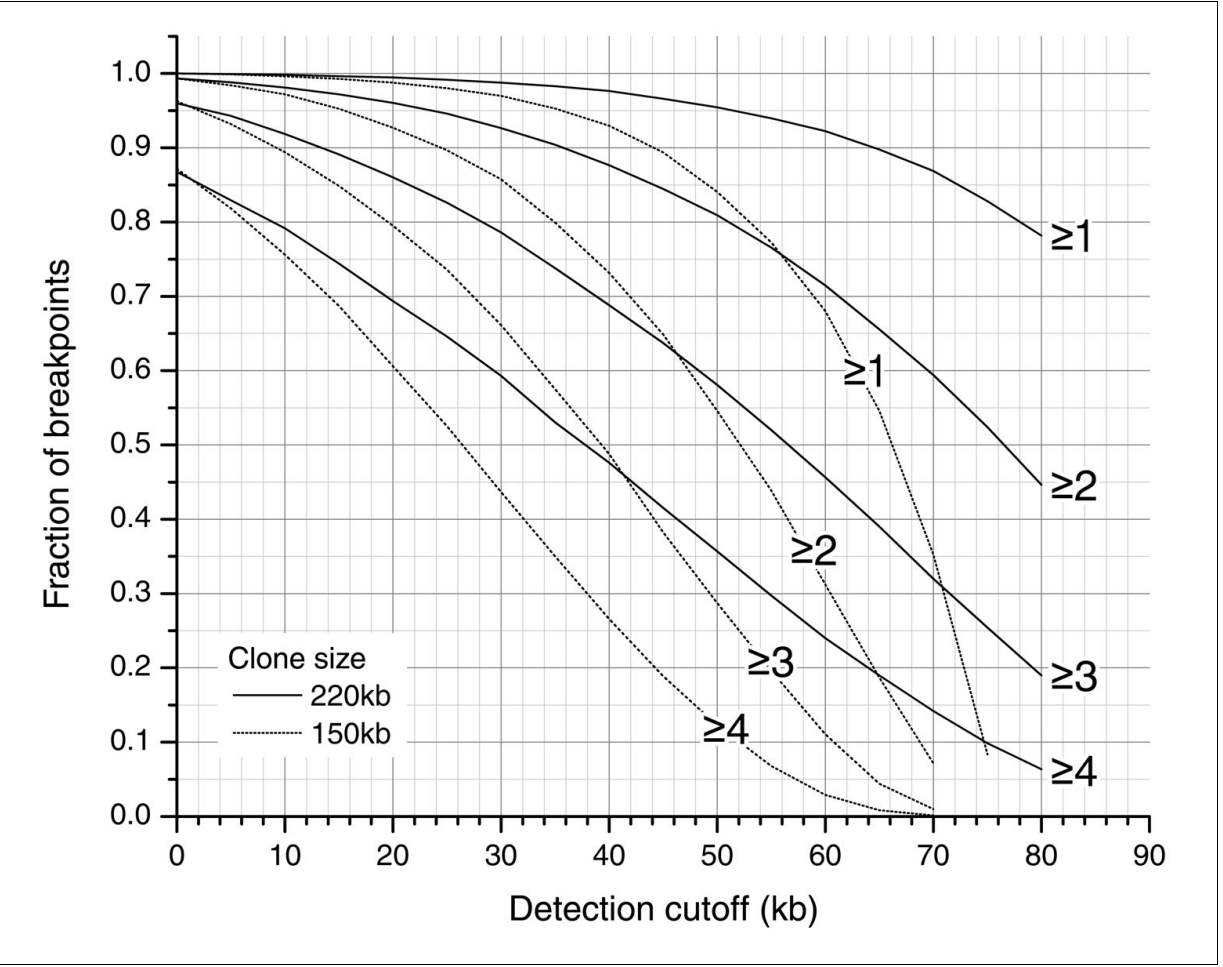
Expected fraction of breakpoints, given five-fold redundant clone coverage, captured by ≥N clones with the distance between breakpoint and clone terminus larger than detection cutoff. The plot shows detection profiles for 150 kb and 220 kb clones. The plot illustrates the benefit of redundant coverage and of using clones with larger inserts - for a given detection cutoff, a breakpoint is captured by significantly more clones on average. The detection sensitivity (Figure 4) needs to be applied to the fraction of breakpoints on this plot (for example, 80% of breakpoints found in ≥2 clones within 50 kb of the ends of the clone; assuming 2 digests, 95% of 50 kb regions can be aligned (Figure 4); therefore, 80% × 0.95 = 76% of breakpoints are expected to be detected in these conditions).

Fingerprint-based alignments confirmed a lack of rearrangement in the vast majority of clones (96%) and also confirmed the presence of rearrangements in 68% of those clones in the test set whose ESP data indicated a breakpoint. The high level of confirmation of clone integrity reflects the low incidence of false-positive alignments for clones derived from a single location. The fraction of rearrangements detected is lower than in ESP due to the inherent limitation of fingerprint-based alignments to align small regions of the genome. The use of larger BACs or greater levels of coverage redundancy (Figure 8) would be expected to address a significant portion of these apparent false-negative FPP results.

A number of studies (reviewed in [31]) have reported on the increasing prevalence of human genome structural alterations in both healthy and diseased individuals. Much of the work has been done using genome-wide microarray technologies, and the median lengths of many of the structural alterations reported are in the range of tens to hundreds of kilobases or more [32]. These lengths correspond to the resolutions possible using the microarray technologies employed for these studies. The resolving power of the FPP approach we report here improves upon the resolution possible with commonly available microarray platforms, and could easily be applied to whole genome characterization. We believe characterization of tens to hundreds of human genome samples using FPP would provide a powerful data set from which to deduce the lengths and types of genome rearrangements in human populations, as well as providing information on the sequences affected and flanking such rearrangements.

## Conclusion

To explore the utility of fingerprint-based rearrangement detection, we used computational simulations and fingerprinted a set of clones derived from the MCF7 breast tumor cell line for which ESP data were available [25]. By collecting multiple fingerprints obtained with different enzymes for each clone and comparing FPP and ESP results for the same clones, we were able to conclude that FPP is well-suited for accurate study of genomic differences. Moreover, we were able to define the boundaries of differences between the reference and MCF7 genomes more precisely than with ESP, and to demonstrate complex rearrangements with FPP that otherwise required BAC shotgun sequencing to fully characterize. Using a set of 493 clones from the MCF7 BAC library sampled primarily to represent content from chromosomes 1, 3, 17 and 20, we used 5 fingerprints to identify 51 breakpoints within the regions sampled by the clones with a median positional error of 2 kb. We were able to reconcile the ESP and FPP data sets and used *in silico *simulations to explore the practical limitations of FPP. Based on our observations, we feel FPP has compelling potential to be used as a whole-genome method to identify and characterize human genome rearrangements.

## Materials and methods

Here we describe the computational and algorithmic components of FPP. The sections broadly comprise generation of target fingerprint patterns and pattern matching, theoretical considerations in generating and using fingerprints for alignment, description of an experimental data set to characterize FPP performance and a detailed description of the FPP algorithm.

### *In silico *simulations: sequence assembly digest

We performed *in silico *simulations to explore the theoretical limitations of using fingerprints to unambiguously identify genomic regions. We used the UCSC May 2004 (hg17) assembly of the human genome for these simulations, using *in silico *digests of sequence assemblies of each chromosome (1-22, X, Y). For each *in silico *digest the size and start/end position for all restriction fragments were calculated and stored. To generate virtual clone fingerprints, groups of adjacent restriction fragments were randomly sampled in accordance with a hypothetical clone size distribution. During the sampling process, we avoided regions of the sequence assemblies that contained undetermined base pairs.

### *In silico *simulations: fingerprint comparison

We calculated similarity between fingerprint patterns using Needleman-Wunsch global alignment [33]. The similarity of two fingerprint patterns was proportional to the number of fragments that were common between fingerprints being compared. Common fragments were defined as fragments whose sizes were equal within measurement error (Additional data file 1). Such fragments have experimentally indistinguishable electrophoretic mobilities. For an estimate of experimental sizing error, we used values obtained from comparing fingerprints of sequenced BAC clones to their computationally predicted counterparts (Figure 3).

### *In silico *simulations: fragment and fingerprint specificity

The degree to which a fingerprint pattern can uniquely represent a genomic region is directly proportional to the efficiency of FPP. See Additional data file 1 for a description of the method used to calculate specificity shown in Figure 2.

### *In silico *simulations: enzyme selection

The choice of restriction enzymes affects the effectiveness of FPP - ideal enzymes are those which cut frequently and in a complementary manner, with cut sites of one enzyme populating regions where another enzyme lacks them. See Additional data file 1 for a description of the simulation performed to select an optimal combination of 5 enzymes.

### *In silico *simulations: generation of virtual clone fingerprints to determine fingerprint-based alignment accuracy

To determine the theoretical performance of fingerprint-based alignment accuracy and the sensitivity and specificity of rearrangement breakpoint detection, we generated *in silico *fingerprint patterns of hypothetical clones derived from a genomic region that contained a simulated breakpoint. To simulate a clone harboring a breakpoint, a fingerprint pattern was created by combining two groups of fragments containing fragments totaling N kb and 180-N kb, derived from randomly sampling two non-overlapping regions of the genome. To simulate the restriction fragment that contained the fusion point, two edge fragments, selected randomly from each group, were combined into a single fragment. We generated 384 180-kb synthetic clones for each value of *N *= 5, 10, 15, 20, 30, 50, 60, 70 and 90 kb and used FPP (see below) to align the fingerprints to the sequence assembly. We quantified the accuracy and detection limits of the alignment method by comparing the alignment results with known clone locations. The positional accuracy of fingerprint-based alignments was evaluated by comparing the difference in position between the fingerprint alignments and the known span of the *in silico *segments of the synthetic clones.

### MCF7 clone test set and end sequencing

We used a subset of BAC clones prepared from MCF7 breast tumor cell line DNA, and identified by S Volik, an author on this study. The average insert size for these clones was 141 kb [25]. We analyzed 493 clones for which paired end sequence alignments to the human sequence assembly (UCSC, hg17) were available [25]. Clone selection was performed by S Volik based on analysis of the alignments of the end sequences to the reference human genome sequence. The set of 493 clones was enriched for clones whose end sequence alignments indicated that the clones identified rearrangements on chromosomes 1, 3, 17 or 20.

### MCF7 clone fingerprinting

We attempted to fingerprint each of the 493 clones as described [34]. Five fingerprints were collected for each clone using the combination of restriction enzymes that was identified as optimal: *Hin*dIII (a|agctt), *Eco*RI (g|aattc), *Bgl*II (a|gatct), *Nco*I (c|catgg) and *Pvu*II (cag|ctg). The average clone size of the test set, based on the average sum of fragments in each fingerprint, was 146 kb. This included the 7.5 kb pECBAC1 vector. We obtained a full complement of 5 fingerprints for 484 of the clones, 4 fingerprints for 6 clones, 2 fingerprints for 2 clones and no fingerprints for 1 clone.

### Fingerprint profiling

The fingerprinted MCF7 clones were mapped to the reference sequence assembly (UCSC, hg17) by aligning their fingerprints against *in silico *fingerprints produced computationally from the assembly. The FPP algorithm is composed of four distinct steps: a global search that broadly identifies BAC-sized (or smaller) regions of the genome that yield digest patterns similar to the clone being aligned; a local search that uses a fragment accounting approach to more precisely delineate the correspondence between fragments found in both the clone fingerprint and the assembly within the boundary of each region; an edge detection algorithm that identifies the extent of the alignment; and a partitioning step that finds a minimal set of alignments that maximally account for all clone fragments on the genome.

### FPP: global search

The fingerprint-based alignment was performed in two steps, first as a global search across the entire genome, followed by a local search. First, the sequence assembly was digested *in silico *with the recognition sequences for the same restriction enzymes used to produce the clone fingerprints. Next, 20 kb regions, spaced every 10 kb, were delineated and *in silico *fragments overlapping a given region (by any fraction of their length) were grouped together into bins. Each region was thus associated with five bins of *in silico *fragments, with each bin composed of fragments from a different enzyme. Clone fingerprints were then compared to patterns formed by binned fragments for the corresponding enzyme. Each region was assigned a similarity score (s_r_) that reflected the similarity between the *in silico *fingerprint and the experimental fingerprint. The individual 20 kb regions were rank-ordered based on their similarity score, and adjacent, possibly overlapping highly scoring regions were grouped together. Region groups were sorted by size and rank-sum.

### FPP: local search and alignment edge detection

A local evidence-based search was performed in the neighborhood of 20 highest ranking grouped regions. The purpose of the local search was to identify more precisely the start and end of the region of the genome whose fingerprint pattern matched the clone's fingerprint. While the global search evaluated similarity for bins spaced every 10 kb across the genome, the local search was sensitive to specific positions of restriction enzyme recognition sequences across all digests. Each clone fingerprint was compared with the corresponding *in silico *fingerprint pattern derived from the area subject to the local search. Fragment covers were defined by the start and end positions of fragments across all digests. Each cover was assigned a score that reflected the extent to which the fragments forming that cover matched the clone fingerprint.

### FPP: alignment edge detection

Once each cover was scored, we used a cumulative evidence model to determine the most likely start and end position of the clone fingerprint alignment. The evidence model used a running sum of cover scores (Additional data file 1) across a region. Covers having low similarity lowered the running sum and covers having high similarity increased the sum. Alignment detection was triggered when the sum grew beyond a cutoff value.

### FPP: identification of minimal set of alignments

To identify the most likely combination of alignments that mapped the clone insert to the genome, we applied a partitioning model based on rules of parsimony. In addition to one or more true-positive alignments, we expected a certain number of false-positive alignments located in regions of the genome with sufficient fingerprint similarity, but distant from the actual points of origin of the clone. To identify these alignments as false-positive, we used the assumption that these alignments were coincidental and, thus, involved clone fragments independent of those involved in the true-positive alignment. To identify the best combination of alignments, we constructed and scored all possible alignment combinations of up to four alignments. For every alignment in a combination, we tabulated the number of fragments that were unique to that alignment (that is, not participating in other alignments in the combination), and fragments found in one or more alignments in the combination. Any combination for which one or more alignments failed the criteria based on unique and redundant alignment content was not considered. Each alignment was required to have at most 20 kb or 2 fragments of redundant content, which could not be more than 20% of the alignment's length. Each alignment was also required to have more than 7.5 kb and 2 fragments of unique content. Combinations composed of alignments that passed were scored on the basis of the reconstruction fraction, defined as the total size of all alignments in the combination relative to the average fingerprint size of the clone. The highest scoring combination of alignments was designated as the real alignment region.

### Comparison of FPP to ESP

Clones with FPP alignments to more than one genome location were considered to harbor a rearrangement. For identifying rearrangements by ESP, we required that the end sequence alignments satisfied one of the following criteria: they had the same orientation (reverse orientation is expected in the normal case); they were separated by more than 500 kb ([25] used the criteria that the observed size be within 3 standard deviations, which was approximately sd = 35 kb, of the library's average insert size); or they aligned to different chromosomes.

ESP data were compared to the FPP alignments to explore the performance characteristics of fingerprint alignments. This comparison was designed to account for the fact that the FPP alignment is a set of one or more spans, while the clone's ESP data are a pair of end sequence alignments that are essentially defined by two points on the genome. Thus, for each of the end sequence alignments, we determined whether there existed an FPP alignment within 50 kb of the end sequence alignment and, if so, the distance between the nearest FPP alignment edge and end sequence alignment. A clone's FPP and ESP data were considered to be in agreement if both end sequence alignments were in the proximity of FPP alignments (Tables 1 and 2).

### Identification of micro-aberrations

Fingerprint-based clone alignments were inspected for evidence of potential small-scale aberrations. Localized regions of incongruence between the reference and MCF7 genomes result in unmatched experimental restriction fragments. Such regions are associated with adjacent covers with a zero, or unusually low, cover score s_c_. The cover score quantifies the level of similarity between the fingerprint patterns of all 5 digests and the in silico pattern of a region of the genome. The cover score is described in greater detail in additional data file 1. We identified these regions by enumerating all unmatched fragments within the FPP alignment bounds for each digest and looking for non-empty intersections of unmatched fragments across all digests.

### Validation of aberrations identified by FPP results

For a subset of clones whose FPP alignments indicated a translocation or a local aberration, we designed PCR primers to establish the presence and nature of the aberration. For gross aberrations, such as translocations, we designed primers to form an amplicon across the breakpoint to demonstrate its presence in the clone. For local aberrations, we designed PCR primers (Additional data file 1) spanning the affected region and sought an amplicon of a size different than suggested by primer placement on the reference genome.

## Abbreviations

BAC, bacterial artificial chromosome; bp, base-pairs; ESP, end sequence profiling; FPP, fingerprint profiling.

## Authors' contributions

MK: data analysis lead, algorithm and software development, manuscript preparation. IB, CM, NW: protocol development, laboratory fingerprint generation. JB: end sequencing and analysis. RC, RC, MF: data analysis. DL: protocol development, laboratory fingerprint generation. TP: PCR and sequencing. SV: ESP data and experiment lead and collaboration. AS, SJ: bioinformatics leads, project management. JS: laboratory lead, project management. MM, CC: principal investigator, laboratory lead, project management. All authors have read and approved the final manuscript.

## Additional data files

The following additional data are available with the online version of this paper. Additional data file 1 provides additional details about the algorithms used to evaluate fingerprint similarity and fragment specificity, to select enzymes, to score fingerprint alignments, to determine alignment edges and to design PCR primers. Additional data file 2 is a figure that shows FPP and BES alignments on regions of chromosomes 1, 3, 17 and 20. Additional data file 3 is a figure that shows a more detailed view of selected regions of chromosomes 1, 3, 17 and 20 from Additional data file 2. Additional data file 4 is a figure that shows a detailed reconciliation of sequence and fingerprint alignments for regions A, C, D and E (Figure 6) of clone 3F05 which contains at least four internal breakpoints. Additional data file 5 is a figure that shows the restriction fingerprint fragment accounting for alignments of 3F05. Additional data file 6 is a figure that shows a high-resolution representation of FPP alignments shown in Additional data file 2.

## Supplementary Material

Additional data file 1 Details about the algorithms used to evaluate fingerprint similarity and fragment specificity, to select enzymes, to score fingerprint alignments, to determine alignment edges and to design PCR primers. Click here for file

Additional data file 2 Regions containing clone alignments are composited horizontally. Alternating vertical white/grey bands indicate disparate regions. Distant alignments of a given clone are joined by horizontal lines. FPP alignments are either black, if validated by BES alignments, or red if no BES alignment is nearby. BES alignments are indicated by small white circles. Blunt termination of FPP alignments is indicative of a breakpoint and these boundaries are denoted by vertical red lines. The boundaries are coded by N(L, R), where N uniquely indexes the breakpoint and L, R indicates whether the breakpoint is on the left or right of the FPP alignment. The breakpoint map generated by FPP alignments is more accurate and complete. FPP alignments captured nearly all breakpoints detected by FPP to a higher degree of spatial accuracy (for example, 3R, 18L). In numerous cases, internal breakpoints were detected by FPP (for example, 7L, 8R). Regions of this figure are presented in greater detail in Figure 7. Click here for file

Additional data file 3 Detailed view of selected regions of chromosomes 1, 3, 17 and 20 from Additional data file 2. Click here for file

Additional data file 4 Restriction maps for each of the five enzymes (*Eco*RI (e) *Hin*dIII (h) *Bgl*II (g) *Nco*I (n) and *Pvu*II (p)) in the neighborhood of regions of M0003F05 detected by FPP. Restriction map fragments matched by experimental fragments in the corresponding fingerprint are shown in green. The FPP alignment is delineated by black vertical lines and the extent of the BLAT alignment is delineated by blue vertical lines. Click here for file

Additional data file 5 Images of individual fingerprints of M0003F05 are accompanied by the image of the nearest marker lane. Fragment sizes are shown to the left of the fragment's band. Correspondence to FPP alignments A, C, D or E (Figure 6) for each fragment is shown. Fragments marked by an asterisk are derived from the digest of the vector. Click here for file

Additional data file 6 The figure depicts the restriction map for each of the five digests used to fingerprint the MCF7 clones. Each clone is shown as a set of five rows, with each row corresponding to the local restriction map of *Eco*RI, *Hin*dIII, *Nco*I, *Bgl*II and *Pvu*II (top to bottom). Fragments within each map are colored green if they were experimentally detected, or red if they were not found in the clone's fingerprint. Adjacent fragments with the same match status are shown with alternating brightness to aid in distinguishing the fragments' extent. Click here for file
