# Supplementary material for: A BAC clone fingerprinting approach to the detection of human genome rearrangements
Source: Genome Biol. 2007 Oct 22;8(10):R224. doi: 10.1186/gb-2007-8-10-r224 (PMC2246298; doi:10.1186/gb-2007-8-10-r224)
Supplement: Additional data file 1 — Details about the algorithms used to evaluate fingerprint similarity and fragment specificity, to select enzymes, to score fingerprint alignments, to determine alignment edges and to design PCR primers. [file gb-2007-8-10-r224-S1.doc]

Supplementary Methods

*In Silico Simulations – Fingerprint Comparison*

Fragments of size $f_{1}$ and $f_{2}$ were considered common if their sizes were within a tolerance value defined at that fragment size, $\left|f_{1}-f_{2}\right|\leq\delta\left(f_{1}\right)$. Since $\delta\left(f\right)$ is much smaller than $f$ and varies slowly with size, for $f_{1}\approx f_{2}$. Our simulations explored two distinct measurement scenarios; exact and experimental sizing tolerance. For the exact measurement case, we employed zero-tolerance, which implies that two fragments were assessed as common only if their sizes were exactly identical. Our experimental tolerance simulations used the sizing error we measured from analysis of sequenced BACs subjected to our laboratory fingerprinting procedure.

*In Silico Simulations – Fragment and Fingerprint Specificity*

We defined the specificity of a single fragment (Figure 2) to be the fraction of genomic fragments that were identical in size and within experimental tolerance, across the entire genome. We computed specificity using exact sizing,$\delta\left(f\right)=0$, as well as experimental tolerance. Single fragment specificity can be interpreted as the probability that the fragment is identical in size by chance with a randomly sampled genomic restriction fragment. To illustrate how the number of fragments in a fingerprint pattern impacts pattern specificity, we studied the occurrence of identical fingerprint patterns with N fragments (Figure 4, N = 3, 4, 5, 6, 7, 10, 15, 20, 30, 40kb). Using in silico digests of chromosome sequence assemblies, we constructed N fragment patterns from digest fragments (i..i+N-1) for each successive fragment position, i. For each successive start position, i, we compared the pattern starting at i to every previously seen pattern and recorded when the pattern was identical to one previously seen. To reflect experimental conditions in the laboratory, the comparison was applied using only fragments within the 0.5-30kb sizing range and fragment sizes were compared using exact or experimental tolerance. Pattern specificity was measured in two ways. First, if a pattern was an exact match to a previous pattern and was derived from a region of the genome that did not overlap with the source of the previous pattern, the pattern was considered to be duplicated in the genome and the size of the pattern was added to a running sum of all duplicated patterns with the same number of fragments. This method produced the size of genomic regions that could not be unambiguously represented by a fragment pattern of size N. Second, we counted the number of individual instances of patterns that appeared two or more times. This method produced a count of all possible non-unique N fragment patterns.

*In Silico Simulations – Enzyme Selection*

We used the in silico digest of the assembly of chromosome 7 (158Mb total size, 155Mb finished sequence, hg17) and characterized 4,060 5-enzyme combinations based on criteria that included: (a) number of fragments in each pattern (more fragments increase the specificity of the pattern, but too many fragments can negatively impact the sensitivity and specificity of fragment detection [24]), (b) the fragment distribution within the pattern (to limit the number of clusters of co-migrating fragments or “multiplets”) and (c) laboratory conditions. Given our experience with the use of restriction enzymes that recognize 6bp sequence sites [24, 28, 29, 35] and the fact that our laboratory process has been tuned to analyzing patterns generated using such enzymes, we limited our selection to enzymes in this class. We further limited our selection to those producing median fragment sizes within the extremes of PstI and XhoI, to avoid the empirical difficulties associated with fingerprints having a large number of fragments (PstI), and low-complexity fingerprints with a small number of fragments (XhoI). The enzymes examined were AclI, AflII, ApaI, ApaLI, AvrII, BalI, BamHI, BclI, BglII, EcoRI, EcoRV, HindIII, HpaI, KpnI, MfeI, NaeI, NarI, NcoI, NdeI, NheI, PmaCI, PstI, PvuII, SacI, ScaI, SmaI, SnaBI, SpeI, SphI, StuI, XbaI and XhoI. To decrease the search space of potential enzyme combinations and focus on combinations that were more likely to yield useful fingerprints, we included HindIII and EcoRI in each combination, based on our experience that these enzymes produce high quality fingerprints based on their robust properties under our experimental conditions and suitable fragment distribution for our analytic conditions [24, 28, 29, 35].

To evaluate the utility of each combination, we determined the proportion of chromosome 7 that was represented in 1-20kb fragments by ≥n restriction fragments in the combination. We defined this quantity as S(n) and sought combinations that maximized S(n) for n=0..5. Since S(n) is a cumulative quantity, S(m) ≥ S(n) for m < n. High values of S(n) were desirable, since they indicated that a large fraction of in silico fragments generated by an enzyme were within a size range of increased sizing accuracy. After computing S(n) for each combination and ranking the results, highly scoring enzyme combinations (Figure 1) were further prioritized on the basis of ease of use in the laboratory, median restriction fragment size and median inter-fragment distance. The inter-fragment distance was defined as the difference in standard mobilities (a standardized unit approximately linear with the distance restriction fragments travel from the gel origin), between adjacent restriction fragments in a fingerprint pattern.

From the characterization of 4,060 restriction enzyme combinations (Figure 1), we identified 132 sets (Figure 1, inset, hollow glyphs) that ranked in the 90th percentile of S(n) (see Supplementary Materials) for each value of n. Each of these 132 combinations, in addition to HindIII and EcoRI, was composed of 3 enzymes from the following subset: AflII, ApaLI, AvrII, BalI, BclI, BglII, NcoI, NdeI, PstI, PvuII, SacI, ScaI, SphI, StuI, and XbaI. Nearly half of the combinations (63/132) had PstI as one of the enzymes, and given the unfavourably high frequency of recognition sequences in the genome, these combinations were discounted. The top four combinations, as ranked by S_ranked_(5) and characterized here by (S(1),S_ranked_(4),S_ranked_(5)), out of the set of 63 which did not contain PstI were: BclI/BglII/PvuII (154.86 Mb, 0.985, 1.000), BalI/BclI/BglII (154.88 Mb, 0.997, 0.997), NcoI/PvuII,XbaI (154.87 Mb, 0.999, 0.996), Bcl/NcoI/PvuII (154.87 Mb, 1.000, 0.994), and BglII/NcoI/PvuII (154.86 Mb, 0.998, 0.994). Coverage of the sampled sequence, given by S(1), was very high for each of these combinations, within 40kb of the maximum S(1) value of 154.89Mb observed for BalI/PstI/PvuII. Each of these combinations ranked highly (>98.5^th^ percentile) based on values of S(4) and S(5). Ultimately, we selected the combination HindIII, EcoRI, BglII, NcoI and PvuII (S(4) 99.8^th^ percentile, S(5) 99.4^th^ percentile) for its desirable cut site distribution, ease of use in the laboratory and our favourable experience with the high quality of fingerprints from these enzymes.

*Fingerprint Profiling (FPP) – Region Similarity Score*

The region similarity score, s_r_, was used during the global search phase of FPP and measures the similarity between the fingerprint pattern of a clone and that of a region of the genome. It is a sum over all digests, d=1..5, and proportional to the number of fragments that matched, m_d_, the total number of fragments, M_d_, and a sum of the log of fragment specificity, p_j_ (see *Fragment and Fingerprint Specificity*).

$$s_{r}=-\sum_{d} \left(\frac{m_{d}}{M_{d}}\sum_{j=1}^{m_{d}} \log p_{j}\right)$$

If all fragments of a region matched, m_d_/M_d_=1, and the score is a reflection of the frequency of these fragments in the genome, appropriately weighted by the sizing tolerance. Large matching fragments, whose specificity is high (Figure 2), contributed more to the score than small fragments. The regions’ similarity score was used to identify genomic locations that were likely to be the points of origin of the clone’s insert.

*Fingerprint Profiling (FPP) – Cover Score*

The cover score, s_c_, was defined by;

$$s_{c}=-\sum_{d} m_{d}\log p_{d}$$

with the sum carried out over d=1..5 digests and m_d_=1 if the fragment was matched and 0 otherwise. As before, p_d_ was the specificity of the cover’s fragment from digest d.

*Fingerprint Profiling (FPP) – Alignment Edge Detection*

The running sum for cover i+1 was $S_{i+1}=S_{i}+f(s_{c})$ where f(s_c_) was an adjusted cover score. The adjustment was designed to grow or shrink the sum based on the value of the cover score. The function f(s_c_) is defined as follows;

$$f(s_{c})=\left\{\begin{matrix} s_{c} \\ -k_{2}s_{c} \\ 0 \end{matrix}\right. \begin{matrix} s_{c}\geq k_{3} \\ s_{c}<k_{3} \\ S(i)+f(s_{c})>k_{4} \end{matrix}$$

The k_2_ factor was a penalty factor and was designed to lower the sum when the cover score, s_c_, was small. The sum was not permitted to grow beyond k_4_. The initial start and end positions of the alignment were determined by the value of S across the covers. Where the sum became larger than k_1_ we deemed the alignment to begin and where it became smaller than k_1_ we deemed the alignment to end. The purpose of using a maximum value of the running sum k_4_ > k_1_ was to allow for one or more fragments to be unmatched before the sum dropped below k_1_. If the sum never grew larger than k_1_ then no alignment was made. By adjusting the values of these parameters the method was made selectively sensitive to runs of high or low scoring covers that indicated the start or end of an alignment. We used 30, -1, 6 and 50 for the parameters k_1_, k_2_, k_3_, and k_4_, respectively. Because S depends on the direction in which the covers are examined, we performed the computation in both directions and obtained two sets of alignment start/end positions. The two sets of positions were averaged by intersecting them to isolate only those portions in which an alignment was made in both directions.

Because the running sum takes several covers to reach k_3_, the alignment typically underestimates the extent of the true alignment if the edges of the region identified by the global search were within a few fragments of the ends of the clone. This compromise in sensitivity was required to avoid triggering alignments composed of only a few matching fragments. To mitigate this, we attempted a final extension of the alignment in which we migrated the alignment regions outwards until we encountered more than two unmatched fragments in any of the digests.

*Validation of Aberrations Identified by FPP Results – Primer Generation*

The PCR primers were selected from 2.5kb flanks on either side of the aberration, as determined by fingerprint-based alignments. Primer 3 [36] was used to select the primer with the following settings: gcclamp = 1, tm_min = 55, tm_opt = 66, tm_max = 70, size_opt = 25, size_min = 24, size_max = 28. Each primer pair was tested with ePCR [37] to ensure absence of multiple products smaller than 15kb.

Validation of clones harboring putative translocations required multiple PCR reactions per clone because the order and orientation of the clone fusion cannot be determined solely from fingerprint data. Given multiple alignment regions of a clone (e.g., A, B, C), a primer was designed from each region end (e.g., left (L) or right (R)) that was not associated with an end sequence alignment. Primers were labeled based on the region/end combination (e.g., AL, BR, etc.) For each primer on the + strand we added its – strand equivalent to the test pool (e.g., AL+ AL-) Various combinations of order/orientation of the fused regions were tested by performing PCR reactions with all possible primer pairs (e.g., AL+/BR+, AL+/BR-, AL-/BR+, etc.).
