# Supplementary figures and images for: A BAC clone fingerprinting approach to the detection of human genome rearrangements
Source: Genome Biol. 2007 Oct 22;8(10):R224. doi: 10.1186/gb-2007-8-10-r224 (PMC2246298; doi:10.1186/gb-2007-8-10-r224)

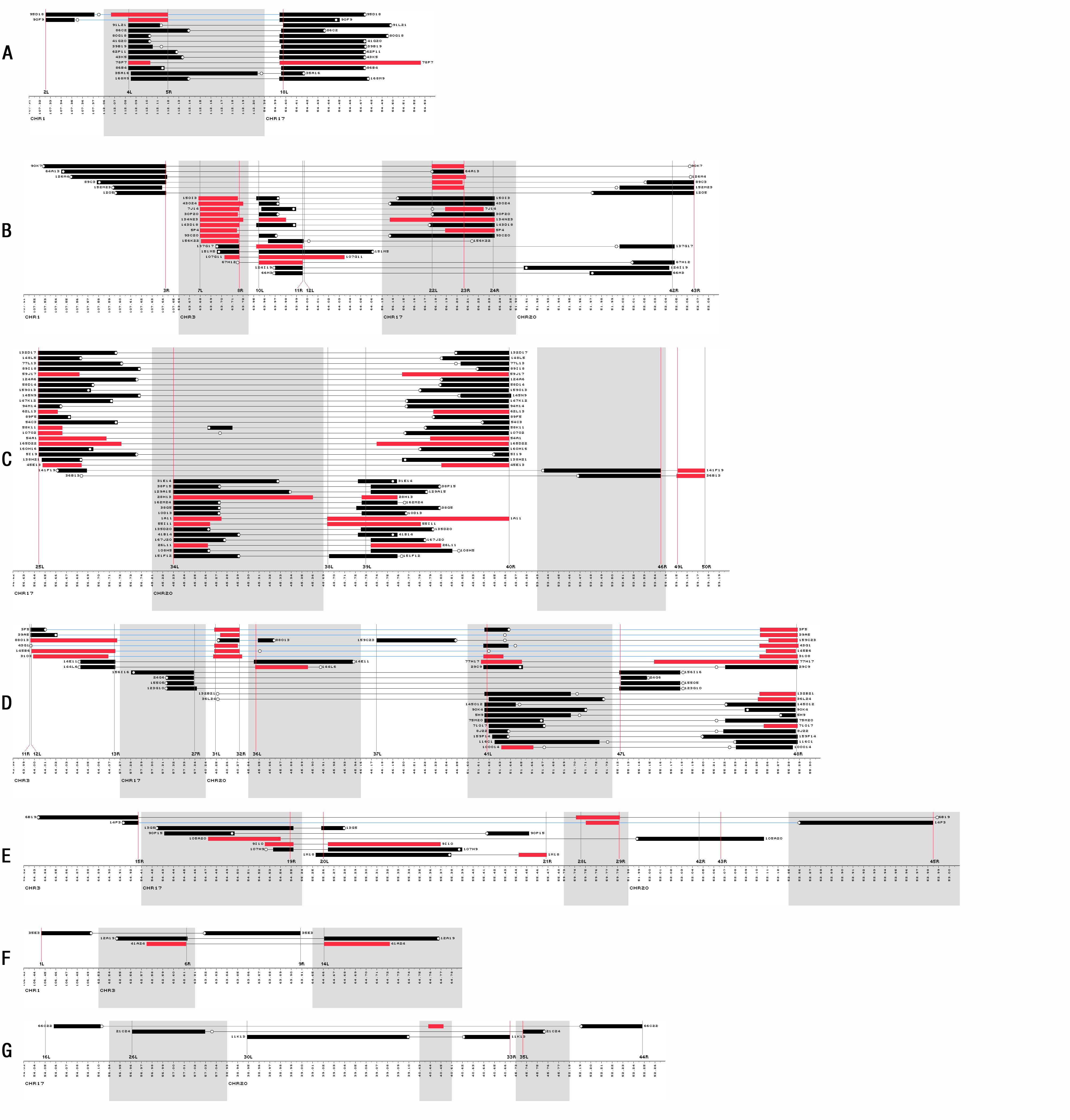

Supplement: Additional data file 3 — Detailed view of selected regions of chromosomes 1, 3, 17 and 20 from Additional data file 2. [file gb-2007-8-10-r224-S3.tiff]

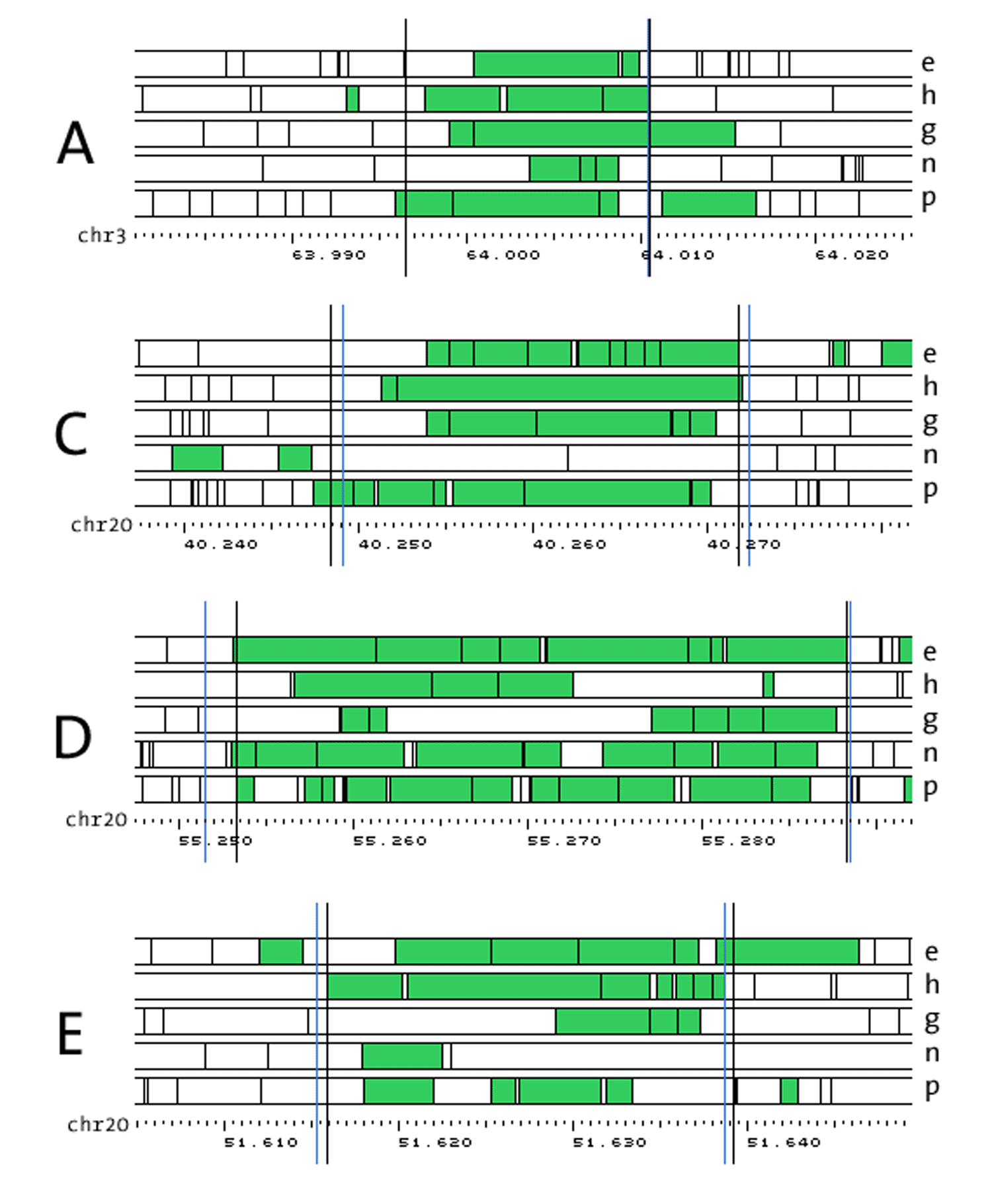

Supplement: Additional data file 4 — Restriction maps for each of the five enzymes (EcoRI (e) HindIII (h) BglII (g) NcoI (n) and PvuII (p)) in the neighborhood of regions of M0003F05 detected by FPP. Restriction map fragments matched by experimental fragments in the corresponding fingerprint are shown in green. The FPP alignment is delineated by black vertical lines and the extent of the BLAT alignment is delineated by blue vertical lines. [file gb-2007-8-10-r224-S4.tiff]

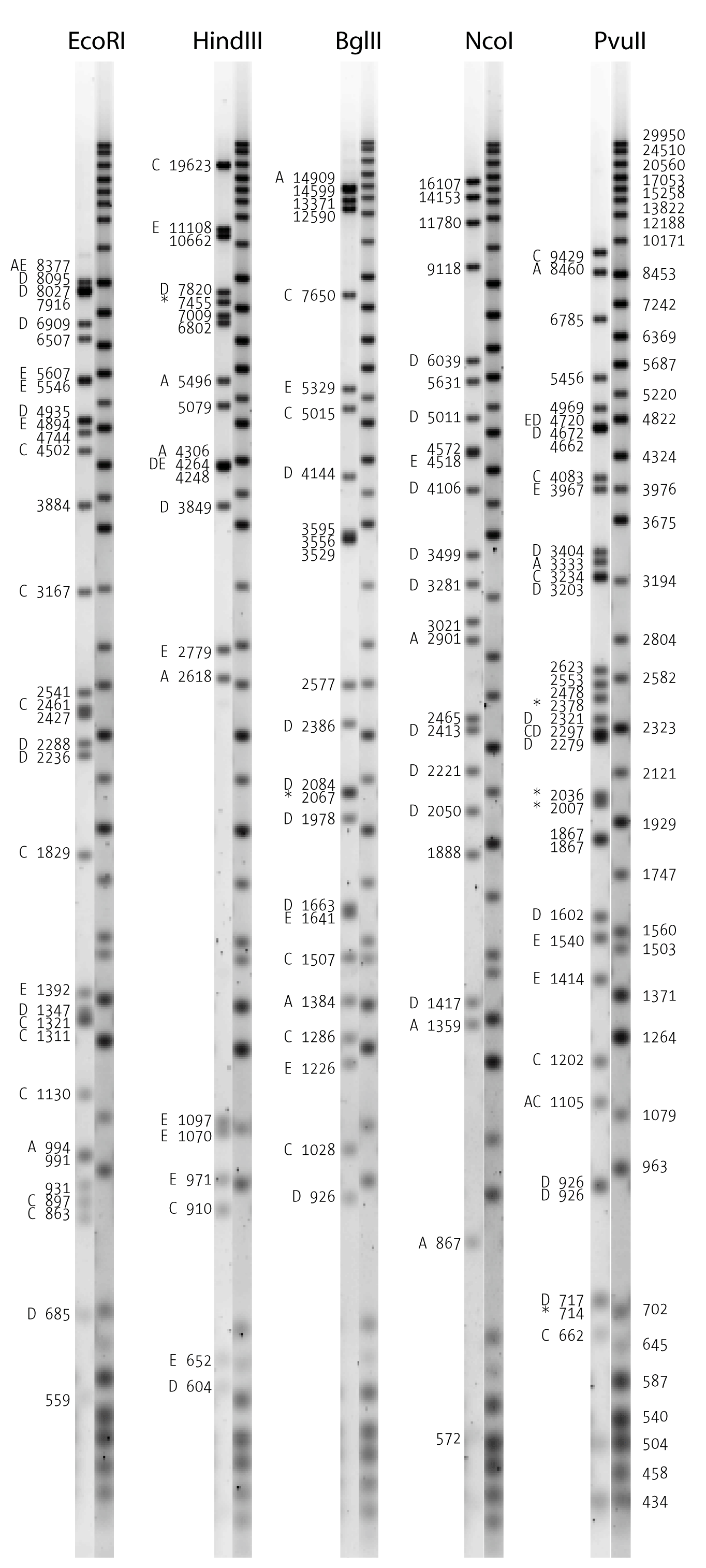

Supplement: Additional data file 5 — Images of individual fingerprints of M0003F05 are accompanied by the image of the nearest marker lane. Fragment sizes are shown to the left of the fragment's band. Correspondence to FPP alignments A, C, D or E (Figure 6) for each fragment is shown. Fragments marked by an asterisk are derived from the digest of the vector. [file gb-2007-8-10-r224-S5.tiff]
